# Rituximab versus natalizumab, fingolimod, and dimethyl fumarate in multiple sclerosis treatment

**DOI:** 10.1002/acn3.51111

**Published:** 2020-08-06

**Authors:** Brandi L. Vollmer, Kavita Nair, Stefan Sillau, John R. Corboy, Timothy Vollmer, Enrique Alvarez

**Affiliations:** ^1^ Rocky Mountain Multiple Sclerosis Center at Anschutz Medical Campus University of Colorado Denver Colorado; ^2^ Skagg’s School of Pharmacy and Pharmaceutical Sciences at Anschutz Medical Campus University of Colorado Denver Colorado

## Abstract

**Introduction:**

Limited comparative effectiveness data for rituximab (RTX) versus natalizumab (NTZ), fingolimod (FTY), and dimethyl fumarate (DMF) for the treatment of multiple sclerosis (MS) exist.

**Methods:**

Clinician‐reported data on patients prescribed RTX, NTZ, FTY, or DMF for the treatment of MS at the Rocky Mountain MS Center at the University of Colorado were retrospectively collected. Outcomes included a composite effectiveness measure consisting of clinical relapse, contrast‐enhancing lesions, and/or new T2 lesions, individual effectiveness outcomes, and discontinuation. Logistic regression was used on patients matched by propensity scores and using average treatment effect on treated doubly robust weighting estimator.

**Results:**

A total of 182, 451, 271, and 342 patients initiated RTX, NTZ, FTY, and DMF and were followed for 2 years. Before and after adjustment, the odds of experiencing disease activity was significantly higher for FTY [adjusted OR (aOR) = 3.17 (95% CI: 1.81–5.55), *P* < 0.001].and DMF [aOR = 2.68 (95% CI:1.67–4.29), *P* < 0.001], and similar for NTZ [aOR = 1.36 (95% CI:0.83–2.23), *P* = 0.216] versus RTX. When examining months 6–24, NTZ demonstrated higher odds of disease activity compared to RTX [aOR = 2.21 (95% CI: 1.20–4.06), *P* = 0.007]. Similar odds of discontinuation were seen between NTZ and RTX [aOR = 1.39 (95% CI: 0.88–2.20), *P* = 0.157]; however, FTY [aOR = 2.02 (95% CI: 1.24–3.30), *P* = 0.005] and DMF [aOR = 3.27 (95% CI: 2.15–4.97), *P* < 0.001] had greater odds of discontinuation than RTX.

**Interpretation:**

RTX demonstrated superior effectiveness and discontinuation outcomes compared to FTY and DMF. Although RTX demonstrated similar effectiveness and discontinuation compared to NTZ, RTX had superior effectiveness during months 6–24 and fewer discontinuations when excluding discontinuations due to insurance issues. Results suggest superiority of RTX in reducing disease activity and maintaining long‐term treatment in a real‐world MS cohort.

## Introduction

Multiple sclerosis (MS) is a chronic inflammatory disease resulting in demyelination and axonal damage in the central nervous system. Current evidence suggests B cells play a role in the pathogenesis, leading to increasing use of anti‐CD20 B‐cell depleting agents in the treatment of MS.^1^ These anti‐CD20 therapies differ in molecular composition and include rituximab and ublituximab (chimeric), ocrelizumab (humanized), and ofatumumab (fully human). Ocrelizumab is the first anti‐CD20 approved for the treatment of both relapsing‐remitting MS (RRMS) and primary progressive MS (PPMS) after three phase three clinical trials.^2^, ^3^ However, prior to this, rituximab, approved in 1997 for non‐Hodgkin’s lymphoma, has been used in the off‐label treatment of MS and is still used today.^4^

Phase 2, double‐blind, clinical trials for rituximab demonstrated a reduction in contrast‐enhancing lesions (CELs) and clinical relapses at 48 weeks in RRMS patients^5^ and has shown a benefit in PPMS for younger patients and those with active inflammatory disease compared to placebo.^6^ Real‐world studies have also deemed RTX to be effective in reducing relapse rates and disability progression in MS patients.^4^, ^7^, ^8^, ^9^, ^10^ While no head‐to‐head randomized clinical trials exist, real‐world studies have conducted comparisons to other highly effective therapies in RRMS patients.^11^, ^12^, ^13^, ^14^

While these studies have provided valuable data for the comparative effectiveness of RTX in RRMS patients, sample size limitations have resulted in conflicting or nonmeaningful results. Furthermore, previous studies were largely conducted in Swedish RRMS populations, limiting generalizability. Our retrospective study aims to address these gaps through achieving a large sample size for investigation of the comparative effectiveness and discontinuation patterns of RTX‐treated patients compared to those treated with NTZ, FTY, and DMF at a large academic center in the United States.

## Methods

### Patient population

This retrospective observational study included all participants who (a) had an MS diagnosis; (b) initiated RTX, NTZ, FTY, or DMF at the Rocky Mountain MS Center at the University of Colorado (RMMSC at CU) between January 2010 and October 2013; and (c) for NTZ patients only, had a negative JCV serology test at baseline. Some JCV positive patients were prescribed NTZ while they transitioned to another DMT. As NTZ was not intended for long‐term care in these cases, we believed they would obscure results and were, therefore, not included. To avoid potential biases resulting from changing documentation practices over time, we limited the data collection for RTX patients to be identical to those of previously collected NTZ, FTY, and DMF cohorts. During the time frame of this study, standard dosing of RTX for the treatment of MS at our center was an induction dose of 2000 mg (1000 mg at day 1 and day 14) and 500 mg every 6 months thereafter in most (77.4%) patients. To be representative of the real‐world experience of MS patients seen in clinical practice, progressive forms of MS were included in this study. However, a subgroup analysis of RRMS patients was completed.

### Data collection

A chart review of electronic medical records was conducted for patients who met inclusion criteria. BV reviewed all RMMSC at CU encounters following each study participant’s start date, defined as date of first administration of RTX, NTZ, FTY, or DMF, for up to 24 months after or until study DMT discontinuation. Baseline characteristics were collected from records at the time of DMT start date. Baseline MRI data were collected from the closest MRI prior to DMT initiation. To confirm accuracy of outliers and consistency of data collection, quality checks were conducted through a second review of a subgroup of charts.

### Outcome measures

The primary outcome was a composite effectiveness measure defined as the patient experiencing a clinical relapse, CEL, or new T2 lesion on follow‐up MRI. Clinical relapses were, for the purpose of this study, defined as clinician‐reported per patient chart notes as new or worsening neurological symptoms lasting greater than 24 h in the absence of fever or infection. MRI data were obtained from neuroradiology reports and clinical reports. At RMMSC at CU, electronic medical records did not consistently capture disability during the time range of this study; therefore, disability was not included in this study.

Secondary outcome measures included (1) individual effectiveness outcomes, including clinical relapse, CELs, and new T2 lesions; (2) discontinuation of therapy, defined as no longer on drug at 24 months after start date, or initiation of any other MS DMT during the 24‐month follow‐up period; (3) primary reason for discontinuation, categorized as disease activity, JCV positivity, AE/tolerability, insurance issues, loss to follow‐up, or any other reason. Some patients withheld therapy for a period of time, for example to alleviate tolerability issues or for travel. However, it was not considered a discontinuation if the patient reinitiated the medication without interruption by any other DMT for the treatment of MS. Patients who developed neutralizing antibodies or had extended dosing intervals were not excluded from analyses as we believe these are characteristics of treatment that affect real‐world effectiveness and ability to achieve long‐term care.

### Statistical analysis

Statistical analyses used SAS Version 9.4 and STATA Version 13.1. R Version 3.1.0 generated Cohen’s D effect size plots.^15^ All two‐tailed *P*‐values < 0.05 were considered statistically significant. Differences in baseline characteristics and secondary outcomes were assessed using *t*‐tests or Wilcoxon ranks sum tests for continuous variables, and chi‐squared or Fischer’s exact tests for categorical data. For the primary outcome and select secondary outcomes, odds ratios (ORs) were calculated using logistic regression. Multiple methods were used to account for imbalances between groups, including simple logistic regression, adjusted logistic regression, logistic regression on sample group 1:2 nearest neighbor matched by propensity scores (PS) with replacement, and average treatment effect on treated (ATT) doubly robust weighting estimator.

Propensity scores generated through logistic regression modeled the probability of receiving RTX treatment using the preselected covariates of age, sex, disease duration, diagnosis, and CEL on baseline MRI. Adjusted logistic regression applied identical covariates as were used in propensity score creation. Kaplan–Meier failure curves assessed cumulative probability of experiencing disease activity, and discontinuation over time. All ORs presented are RTX‐treated patients compared to those treated with NTZ, FTY, or DMF, individually. Comparisons between NTZ, FTY, and DMF have been previously published.^16^, ^17^ Additional analyses investigated outcomes for the RRMS, disease activity during months 6‐24, and discontinuations overall excluding insurance issues. We were unable to adequately match RRMS using propensity matching 1:2 nearest neighbor with replacement; therefore, this method was excluded from the RRMS analysis. All other methods of adjustment for RRMS are presented, including ATT doubly robust weighting using propensity scores.

## Results

### Baseline characteristics

**Figure 1:**
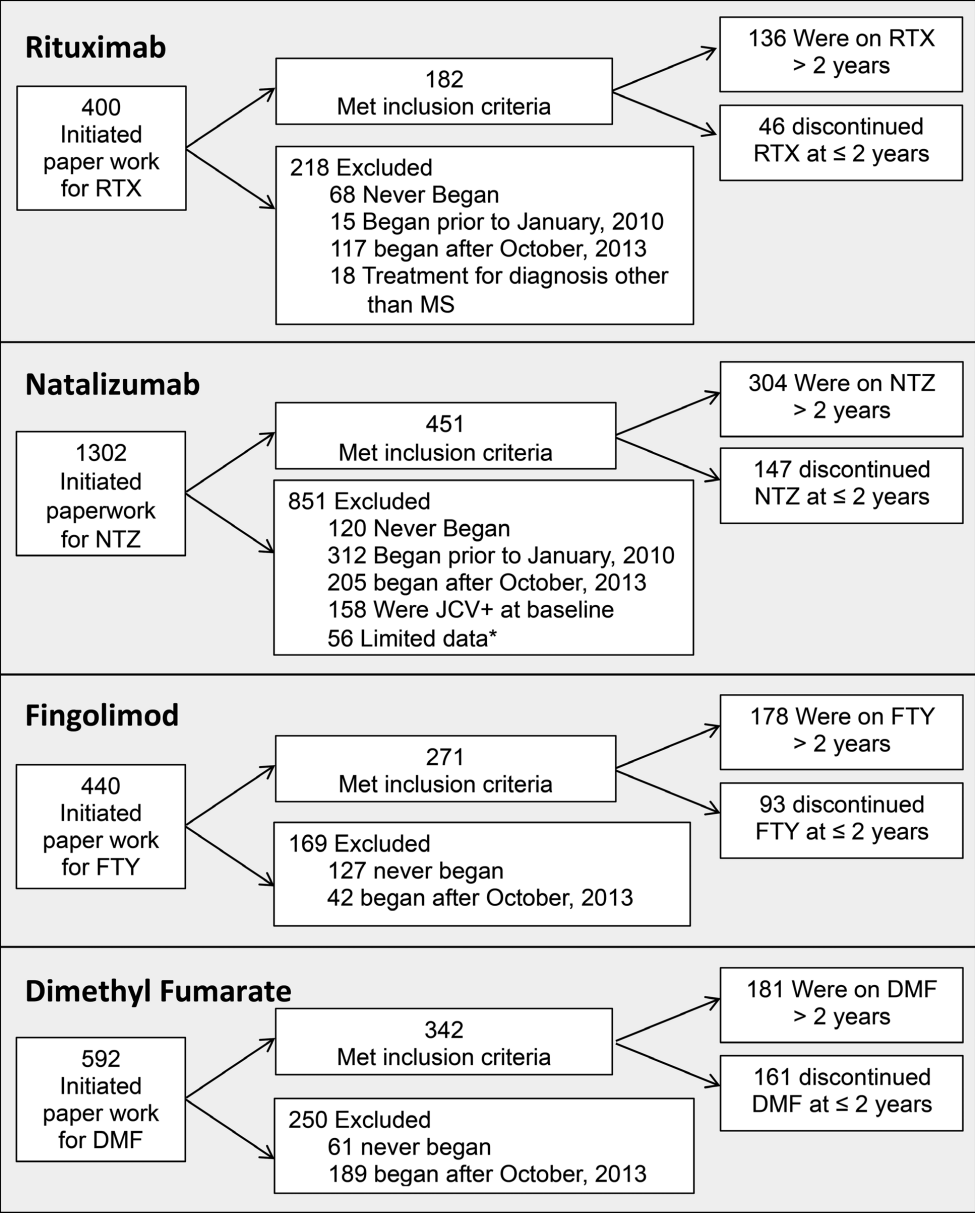
Sample identification. *Limited data refers to patients who transferred to our center after already initiating study drug with no medical records documenting the first two years of treatment and patients who participated in research studies resulting in limited access to data for this study. RTX: rituximab; NTZ: natalizumab; FTY: fingolimod; DMF: dimethyl fumarate.

A total of 1,246 participants met inclusion criteria for this study: 182 RTX, 451 NTZ, 271 FTY, and 342 DMF. Figure 1 shows the overall study flow. Table 1 exhibits baseline characteristics for each study cohort compared to RTX.

**Table 1:** Baseline characteristics for rituximab (RTX), natalizumab (NTZ), fingolimod (FTY), and dimethyl fumarate (DMF) study cohorts.

	Rituximab (*N* = 182)	Natalizumab (*N* = 451)		Fingolimod (*N* = 271)		Dimethyl Fumarate (*N* = 342)	
	*N* or Mean (SD or %)	*N* or Mean (SD or %)	*P*‐value^1^	*N* or Mean (SD or %)	*P*‐value^1^	*N* or Mean (SD or %)	*P*‐value^1^
Disease duration (Years, SD)	12.7 (8.4)	11.4 (7.5)	0.064	11.5 (7.5)	0.128	11.1 (7.4)	**0.028**
Age (Years, SD)^2^	43.9 (11.8)	39.8 (12.1)	**<0.001**	42.5 (11.4)	0.214	45.8 (12.2)	0.078
Sex – Female	120 (65.9%)	346 (76.7%)	**0.005**	195 (72.0%)	0.172	238 (69.6%)	0.392
Type of multiple sclerosis			**<0.001**		**<0.001**		**<0.001**
Relapsing‐remitting	113 (62.1%)	382 (84.7%)		244 (90.0%)		265 (77.5%)	
Secondary progressive	41 (22.5%)	58 (12.9%)		23 (8.5%)		54 (15.8%)	
Primary progressive	28 (15.4%)	11 (2.4%)		4 (1.5%)		23 (6.7%)	
Previous DMT^3^			**<0.001**		**<0.001**		**<0.001**
Interferons	5 (2.8%)	106 (23.5%)		36 (13.3%)		49 (14.3%)	
Glatiramer acetate	13 (7.1%)	152 (33.7%)		49 (18.1%)		106 (31.0%)	
Natalizumab	85 (46.7%)	0 (0.0%)		115 (42.4%)		65 (19.0%)	
Rituximab	0 (0.0%)	1 (0.2%)		1 (0.4%)		9 (2.6%)	
Fingolimod	25 (13.7%)	8 (1.8%)		0 (0.0%)		24 (7.0%)	
Dimethyl fumarate	0 (0.0%)	2 (0.4%)		1 (0.4%)		0 (0.0%)	
None	51 (28.0%)	170 (37.7%)		66 (24.4%)		84 (24.6%)	
Other	3 (1.7%)	12 (2.7%)		3 (1.1%)		5 (1.5%)	
Contrast Enhancement on Baseline MRI^4^	48 (28.4%)	123 (33.1%)	0.280	57 (24.6%)	0.389	44 (14.6%)	**<0.001**
Disease Burden on Baseline MRI^5^			**<0.001**		**0.001**		**<0.001**
Mild	53 (29.1%)	195 (43.2%)		100 (36.9%)		170 (49.7%)	
Moderate	68 (37.4%)	132 (29.3%)		76 (28.0%)		94 (27.5%)	
Severe	45 (24.7%)	34 (7.5%)		45 (16.6%)		29 (8.5%)	
Missing	16 (8.8%)	90 (20.9%)		50 (18.5%)		49 (14.3%)	

Bold *P*‐values indicate *P*> 0.05 and are considered statistically significant.

^1^ In comparison to RTX.

^2^ Median (interquartile range): RTX = 44 years (36‐51) NTZ = 40 years (31–48) FTY = 43 years (35–51) DMF = 47 years (38–55)

^3^ Within 6 months prior to starting study drug.

^4^ Percentage calculated using denominator as those who had baseline MRI with contrast data (RTX N = 169; NTZ *N* = 372; FTY *N* = 232; DMF *N* = 302) .

^5^ Disease burden at baseline is defined as mild < 10 T2/Flair Lesions, moderate 10‐20 T2/FLAIR lesions, severe> 20 T2/FLAIR lesions

### Propensity model

Cohen’s D values for effect sizes comparing baseline covariates between RTX and NTZ/FTY/DMF (Figure S1) demonstrate treatment groups are poorly balanced prior to adjustment with a majority of covariates having absolute standardized differences greater than 10% (absolute standardized difference of the linear PS, comparing NTZ vs. RTX = 76%, FTY vs. RTX = 80%, DMF vs. RTX = 67%). However, we achieve well‐balanced groups through application of ATT doubly robust weighting, with no covariates having an absolute standardized difference greater than 10% for RTX versus NTZ and RTX versus DMF and a linear PS distribution standardized difference of 4.2% and 3.4%, respectively. While RTX versus FTY have one covariate greater than 10% after ATT doubly robust weighting, the linear PS distribution has a standardized difference of 0.1%, well within the 50% standard proposed by Rubin.^18^

### Effectiveness outcomes

**Figure 2:**
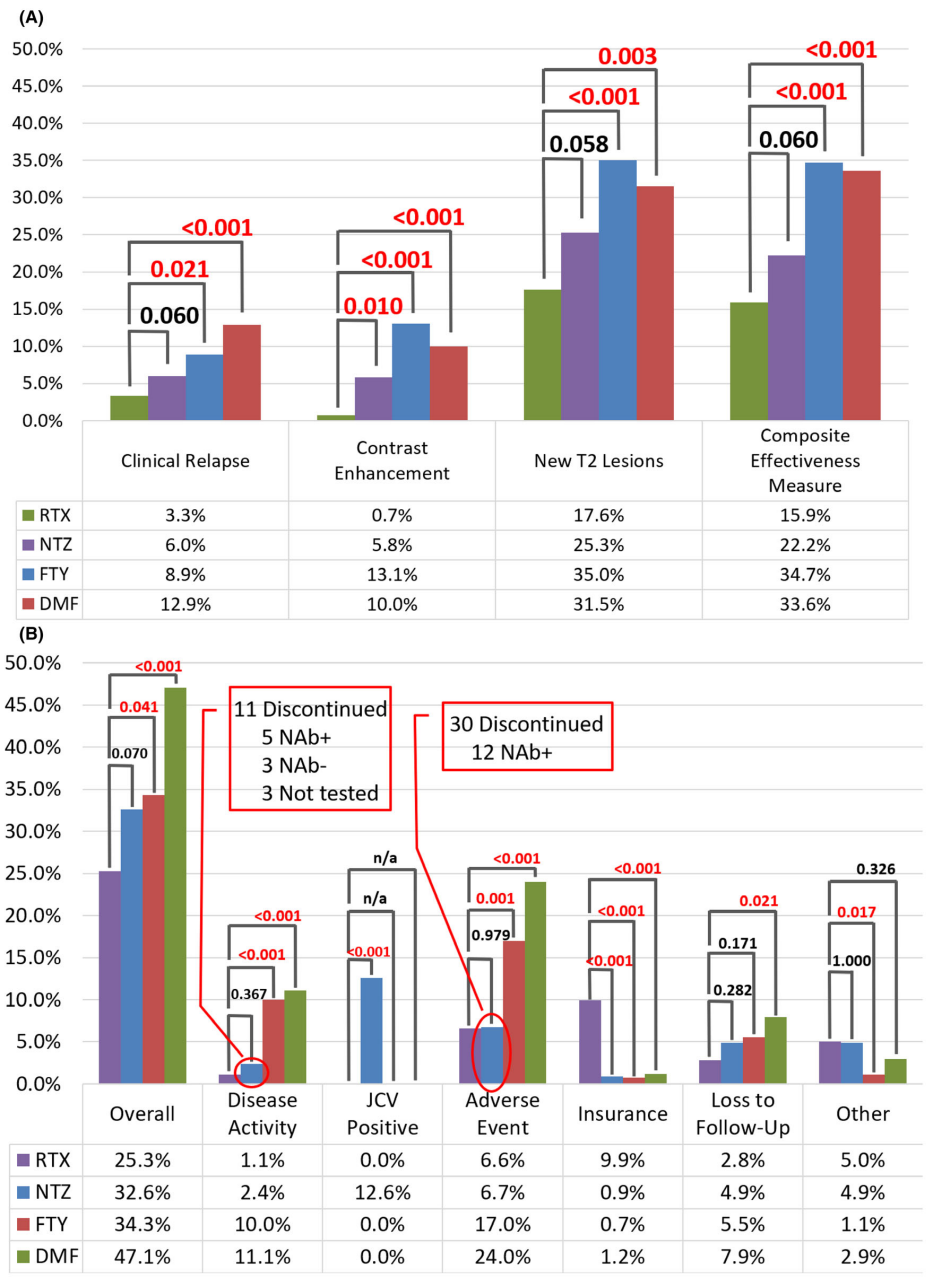
(A) Unadjusted Effectiveness Outcomes (B) Unadjusted Discontinuation Outcomes. RTX: rituximab; NTZ: natalizumab; FTY: fingolimod; DMF: dimethyl fumarate; NAb: neutralizing antibody Composite Disease Activity Measure is comprised of clinical relapse, contrast enhancement and/or a new T2 lesion.

Figure 2A demonstrates unadjusted comparisons. After adjustment, there is no difference in odds of patients experiencing a clinical relapse, CEL, and/or new T2 lesion for NTZ versus RTX (Table 2). Of the 26 NTZ patients tested for NTZ‐neutralizing antibodies, 17 tested positive, 7 of which experienced disease activity. Additionally, 26 NTZ patients have at least one interval between doses greater than 1.5 months, 11 of which experienced disease activity. For FTY versus RTX after adjustment, there is greater odds of FTY patients experiencing a clinical relapse, CEL, and/or new T2 lesion [OR = 3.17 [95% CI (1.81–5.55), *P* < 0.001]. After adjustment, results for DMF versus RTX demonstrate greater odds of DMF patients experiencing a clinical relapse, CEL, and/or new T2 lesion [OR = 2.68 [95% CI (2.68‐4.29), *P* < 0.001].

**Table 2:** Unadjusted and adjusted odds ratios for disease activity regardless of discontinuation in a composite effectiveness measure (new T2 lesion, CEL, and/or new clinical relapse) at ≤ 24 months.

	NTZ vs. RTX			FTY vs RTX			DMF vs RTX		
	*N*	Odds Ratio (95 % CI)	*P*‐value	*N*	Odds Ratio (95 % CI)	*P*‐value	*N*	Odds Ratio (95 % CI)	*P*‐value
Simple logistic regression	633	**1.54** (0.98, 2.43)	0.062	453	**2.85** (1.78, 4.55)	**<0.001**	524	**2.71** (1.72, 4.27)	**<0.001**
Adjusted logistic regression^1^	633	**1.44** (0.89, 2.31)	0.136	453	**3.16** (1.88, 5.30)	**<0.001**	524	**3.04** (1.85, 5.00)	**<0.001**
PM 1:2 NN with replacement^1^	546 (388 Unique)	**1.71** (0.98, 2.96)	0.057	546 (347 unique)	**2.56** (1.40, 4.68)	**0.002**	546 (371 unique)	**3.30** (1.90, 5.71)	**<0.001**
ATT doubly robust weighting estimator^1^	633	**1.36** (0.83, 2.23)	0.216	453	**3.17** (1.81, 5.55)	**<0.001**	524	**2.68** (1.67, 4.29)	**<0.001**

RTX, rituximab; NTZ, natalizumab; FTY, fingolimod; DMF, dimethyl fumarate; CEL, contrast enhancing lesion; CI, confidence interval; PM, propensity matching; NN, nearest neighbor.

Bold *P*‐values indicate *P*> 0.05 and are considered statistically significant.

^1^ Controlling for age, sex (female/male), disease duration, diagnosis (relapsing‐remitting MS/secondary progressive MS/primary progressive MS) and CEL on baseline MRI (yes/no/no MRI available).

**Figure 3:**
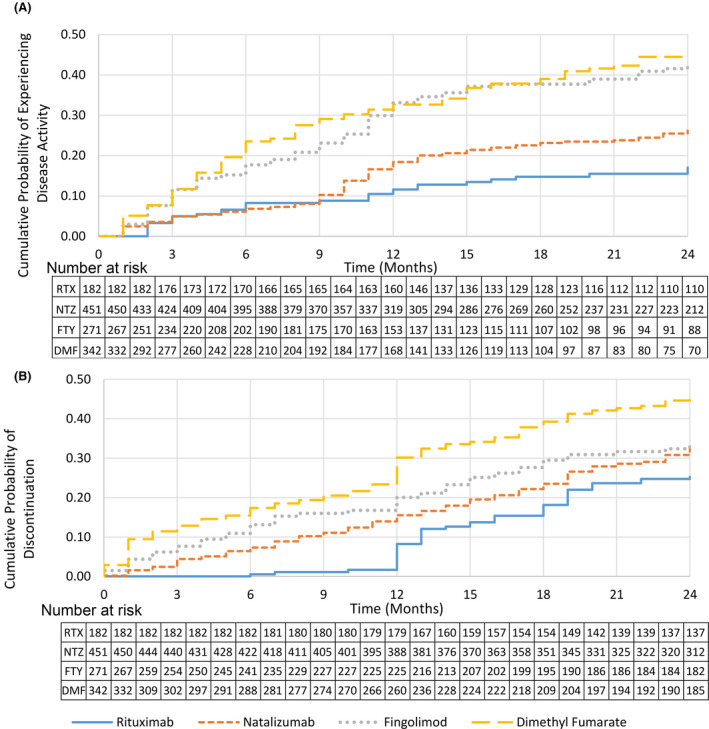
Kaplan–Meier failure curves demonstrating (A) Cumulative probability of experiencing disease activity over time, including clinical relapse, contrast enhancing lesion and/or new T2 lesion, (B) cumulative probability of discontinuation for any reason over time.

Figure 3A exhibit the Kaplan–Meier failure curve demonstrating cumulative probability of experiencing a clinical relapse, CEL, and/or new T2 lesion. RRMS‐only patients demonstrate results consistent with the overall cohort as seen in Tables S1 and S2. When investigating disease activity between months 6 and 24, adjusted results for the composite effectiveness measure are consistently significant (Table S4) for DMF versus RTX and FTY versus RTX. However, while results for the composite effectiveness measure are insignificant for the overall NTZ versus RTX cohort in months 0‐24, there is significantly greater odds of NTZ patients experiencing a clinical relapse, CEL, and/or new T2 lesion between months 6 and 24. Time to event analyses demonstrate consistent results for the composite effectiveness measure after adjustment as the overall cohort (Table S5).

### Discontinuation outcomes

Figure 2B presents the unadjusted discontinuation outcomes overall and by reason. The most common reasons for discontinuation that are categorized as “other” include preference for no DMT and pregnancy for RTX patients, pregnancy, and preference for a more convenient DMT for NTZ patients and nonadherence and attempting pregnancy for both FTY and DMF patients.

Table 3 presents odds ratios for discontinuation due to any reason ≤ 24 months. All methods of adjustment demonstrate consistent results. No significant difference is observed for NTZ versus RTX, while FTY and DMF patients have greater odds of discontinuation [FTY: OR = 2.02, 95% CI (1.24–3.30), *P* = 0.005; DMF: OR = 3.27, 95% CI (2.15–4.97), *P* < 0.001 using ATT doubly robust weighting]. RRMS‐only patients demonstrate results consistent with the overall cohort as seen in Table S3. Figure 3B, and Figures S2 and S3 exhibit Kaplan–Meier failure curves demonstrating cumulative probability of discontinuation overall, discontinuation overall excluding reasons for insurance, and discontinuation due to adverse events. Table S6 and S7 show odds ratios for discontinuation overall, excluding reasons for insurance, and adverse events. Of those who tested positive to NTZ‐neutralizing antibodies, the mean time to discontinuation was 7.5 months.

**Table 3:** Unadjusted and adjusted odds ratios for discontinuation for any reason at ≤ 24 months.

	NTZ vs RTX			FTY vs RTX			DMF vs RTX		
	*N*	Odds Ratio (95 % CI)	*P*‐value	*N*	Odds Ratio (95 % CI)	*P*‐value	*N*	Odds Ratio (95 % CI)	*P*‐value
Simple logistic regression	633	1**.43** (0.97, 2.11)	0.071	453	**1.54** (1.02, 2.35)	**0.042**	524	**2.63** (1.77, 3.91)	**<0.001**
Adjusted logistic regression^1^	633	**1.38** (0.90, 2.13)	0.146	453	**1.96** (1.23, 3.13)	**0.005**	524	**3.32** (2.15, 5.13)	**<0.001**
PM 1:2 NN with replacement^1^	546 (372 Unique)	**1.18** (0.70, 2.01)	0.535	546 (347 unique)	**2.96** (1.64, 5.32)	**<0.001**	546 (371 unique)	**3.34** (2.02, 5.50)	**<0.001**
ATT doubly robust weighting estimator^1^	633	**1.39** (0.88, 2.20)	0.157	453	**2.02** (1.24, 3.30)	**0.005**	524	**3.27** (2.15, 4.97)	**<0.001**

RTX, rituximab; NTZ, natalizumab; FTY, fingolimod; DMF, dimethyl fumarate; CEL, contrast enhancing lesion; CI, confidence interval; PM, propensity matching; NN, nearest neighbor.

Bold *P*‐values indicate *P*> 0.05 and are considered statistically significant.

^1^ Controlling for age, sex (female/male), disease duration, diagnosis (relapsing‐remitting MS/secondary progressive MS/primary progressive MS) and CEL on baseline MRI (yes/no/no MRI available).

### Adverse Events/Tolerability

Tables S7 and S8 displays the odds ratios for discontinuations due to AEs for NTZ/FTY/DMF versus RTX and the type of AE resulting in discontinuation by therapy. Infections are the most common AE leading to discontinuation of RTX, accounting for 2.7% of all RTX patients. For NTZ patients, the most common AE cited as a reason for discontinuation is flushing, rashes, or hot flashes (3.1%), and for both FTY and DMF patients, is GI‐related issues (FTY: 4.1%; DMF: 19.3%).

## Discussion

In this retrospective, real‐world study, we investigated the comparative effectiveness and discontinuation patterns for MS patients treated with RTX in comparison with those treated with NTZ, FTY, and DMF, individually. We achieved large sample sizes, and additionally, were able to create well‐balanced groups for comparisons through the utilization of PS adjustment. Results demonstrated improved effectiveness outcomes and decreased odds of discontinuation for RTX compared with FTY and DMF. While similar effectiveness and odds of discontinuation were observed for RTX compared with NTZ in the overall cohort for months 0‐24, we saw increased effectiveness of RTX when examining months 6‐24.

Our results suggest improved effectiveness of RTX over NTZ when accounting for time to therapeutic effect. Potential explanations for this may be missed or delayed NTZ doses and/or neutralizing antibodies may result in increased disease activity after 6 months for those treated with NTZ. While previous studies took time to therapeutic effect into account, including effectiveness outcomes occurring at least 3 months after the first DMT dose, they have struggled with investigating comparative effectiveness of NTZ compared with RTX in a meaningful way due to smaller sample sizes.^11^, ^12^ Granqvist et al. found a significant difference in clinical relapses between NTZ versus RTX prior to adjustment using sample sizes of 50 NTZ and 150 RTX.^11^ However, after adjustment, the difference became insignificant. Additionally, Boremalm et al. found no difference between clinical relapses for NTZ and RTX before and after adjustment with sample sizes of 105 NTZ and 48 RTX and a mean follow‐up time of 2.8 years.^12^

When comparing FTY to RTX, our results were consistent with previous studies showing improved effectiveness of RTX. Alping et al and Boremalm et al demonstrated superiority of RTX over FTY in RRMS for clinical relapse and CEL outcomes after adjustment.^12^, ^13^ Similarly, our study revealed a significant difference for individual outcomes of clinical relapse, CELs, and new T2 lesions, in addition to our composite effectiveness measure. While Granqvist et al did not see significant differences for their comparisons to FTY after adjustment, this is likely due to their small FTY sample size of *n* = 17.^11^

Currently, to our knowledge, Granqvist et al has conducted the only other comparison of DMF versus RTX using real‐world data and found those treated with RTX had fewer new CELs, but clinical relapses were similar between the two therapies.^11^ These results conflict with our study as we saw a difference among all our individual effectiveness measures, as well as our composite effectiveness measure. However, this may result from differences in sample sizes.^11^

Additionally, while these studies conducted by Granqvist et al, Boremalm et al, and Alping et al include both clinical relapses and MRI outcomes, a composite measures may be required when investigating efficacy among highly effective DMTs, allowing for increased power needed to detect smaller differences through observation of more events.^11^, ^12^, ^13^ This is further supported by the increased utilization of no evidence of disease activity (NEDA) in MS studies, defined as no relapses, no disability progression, and no MRI activity (new or enlarging T2 lesions or CELs).^19^, ^20^, ^21^

When investigating discontinuation outcomes, we found lower odds of discontinuation of RTX compared to FTY and DMF, consistent with previous studies.^11^, ^12^, ^13^ Although odds of discontinuation of RTX were similar to NTZ, RTX discontinuations were driven by insurance issues, as off‐label use poses a challenge for achieving coverage in the United States. Importantly, when examining discontinuations excluding issues with insurance/cost, RTX has significantly lower odds of discontinuation. In this way, superiority of RTX over NTZ in DMT persistence are consistent with Granqvist et al and Boremalm et al, two studies conducted in Swedish populations.^11^, ^12^ Unlike in the United States, national health insurance in Sweden covers all DMTs, including off‐label therapies, therefore, insurance was not a contributing factor to discontinuations. In cases where barriers due to insurance coverage can be overcome, RTX treatment with twice a year infusions shows promise in achieving long‐term efficacy and improved adherence, which will also likely contributed to better disease outcomes.^22^, ^23^, ^24^

Meanwhile, FTY and, particularly, DMF are known to have issues with tolerability and AEs. This, in combination with the daily administration required of these oral therapies has a probable effect on adherence.^25^, ^26^ Furthermore, FTY and DMF discontinuations appear to be driven by AEs, limiting the achievement of long‐term treatment. While few RTX patients discontinued for this reason, the odds were similar to that of NTZ patients. Additionally, the proportion of RTX patients to discontinue due to AEs is higher in our study compared to previously conducted Swedish studies investigating comparative effectiveness.^11^, ^12^, ^13^ This may be due to inclusion of an older RTX population compared to that of other studies (Median age: 44 vs. 37.8 and 39.1 years), as age has been associated with increased risk of infections.^27^

In addition, due to the off‐label use of RTX in the treatment of MS, dosing strategies have varied, which may affect likelihood of AEs and potential benefit. Previous Swedish comparative studies investigated cohorts who similarly received 500‐mg every 6 months, but with few receiving an initial dose of 2000 mg (1000 mg at day 1 and day 14), as was common at our center during the time frame of this study. However, another Swedish study investigating exclusively RTX patients (*n* = 822), of which 32.6% received an induction dose of 2000mg, included both relapsing and progressive patients with a mean age of 42.6 and demonstrated discontinuations due to AEs to be 5.2% (mean follow‐up time 21.8 months), similar to the 6.6% in our study.^4^ Reducing the induction dose to 1000 mg or 500 mg may reduce AEs, and maintain effectiveness as previous studies have shown no significant difference in B‐cell reconstitution at 6 months after induction doses of 1000 mg and 2000 mg.^28^ While a 2000 mg induction dose was representative of our clinic practices at the time of this study, clinicians at RMMSC typically employ a single RTX induction dose of 500 mg today.

Due to the nonrandomized, retrospective nature of this study, there were inherent methodological limitations. Disability outcomes were not available. Follow‐up was for two years, potentially enhancing short‐term benefits or obscuring discontinuations due to infections or other AEs seen with longer term follow‐up. Although adjustment methods appeared to achieve well‐balanced groups, our results may be confounded by indication or unmeasured covariates. However, covariates included in adjustment methods, we believe, are largely representative of characteristics used in clinical practice during DMT decision‐making. For our study, adherence was not thoroughly examined or adjusted for in our analyses, which could impact results for disease activity. As adherence may be affected by frequency of administration and tolerability issues, which vary by therapy, the impact of nonadherence may not be systematic throughout our study. However, as we are investigating real‐world effectiveness rather than efficacy, we believe adherence is a characteristic of each therapy affecting real‐world patients. Therefore, it was not adjusted for in analysis, but rather is a benefit of therapies with reduced frequency of administration and improved tolerability in achieving low disease activity. Furthermore, this study was conducted at a single large academic center, possibly limiting generalizability. Clinicians may differ in DMT prescribing practices and counseling. Additionally, unlike most other studies, we included progressive patients who were older and had lower risk of new inflammatory disease activity such as relapses or MRI scan lesions, which, although this is more representative of patients seen in clinical practice, may influence outcomes. To overcome this, we included an RRMS subgroup analysis, confirming results for the overall cohort. Finally, as a retrospective study, all MRIs were standard of care. As a result, MRIs were not obtained consistently at routine intervals and differing magnetic strength may have been used. This could potentially affect the likelihood of detecting a new T2s or CELs. However, we believe the inclusion of MRI outcomes provides critical information regarding the efficacy of highly effective therapies, and sufficient scans were obtained to provide meaningful information.

In conclusion, our study provided valuable class III information for achieving effective, long‐term care in the treatment of MS. RTX was superior to FTY and DMF, with regard to real‐world effectiveness, tolerability, and DMT persistence. FTY and DMF patients were more likely to discontinue due to adverse events and disease activity. While RTX had similar effectiveness overall compared to NTZ, NTZ was inferior when excluding events during the first 6 months of treatment to allow for time to therapeutic effect. This difference in real‐world effectiveness was likely driven by NTZ‐neutralizing antibodies and missed or delayed doses due to monthly infusions required for NTZ treatment, while RTX was administered twice a year, rather than differences in efficacy between the two therapies. Additionally, although similar to NTZ for overall discontinuations, insurance issues drove RTX discontinuations, while JCV seroconversion contributed largely to discontinuation of NTZ. Further long‐term studies are needed to investigate rare serious adverse events and their risk factors for patients treated with these highly effective therapies.

## Conflicts of Interest

Brandi Vollmer has nothing to disclose. Kavita V Nair has consulted and/or received research support from Genentech, Novartis, Biogen, and Celgene. Stefan Sillau has nothing to disclose. John R Corboy has received grant support from Novartis, Med Day, NMSS, and PCORI; sits on a steering committee for a clinical trial with Novartis; consults with Mylan on a legal issue; receives honorarium for speaking from the Rocky Mountain MS Center and PRIME CME, and receives compensation as editor of Neurology Clinical Practice. Timothy Vollmer has received compensation for activities such as advisory boards, lectures, and consultancy with the following companies and organizations: Biogen IDEC, Genentech/Roche, Siranax, Celgene, EMD Serono, and Novartis. Enrique Alvarez has consulted for Actelion/Janssen, Bayer, Biogen, Celgene, EMD Serono, Genentech, Genzyme, Novartis, and TG Therapeutics; and received research funding from Rocky Mountain MS Center, Biogen, Genentech, Novartis, and Patient‐Centered Outcomes Research Institute.

## Supporting information


**Data S1.** SupplementalRTXCOMP.pdf. Supplemental tables and figures examining Cohen’s D values for effect sizes comparing baseline covariates (Figure S1), outcomes for the relapsing‐remitting multiple sclerosis patients only (Table S1‐S3), disease activity during months 6‐24 (Table S4), time to event analyses for effectiveness (Table S5), discontinuations overall excluding insurance issues (Table S6 and Figure S2), discontinuations due to adverse events (Table S7 and Figure S3), and types of adverse events (Table S8).

## References

[1] Hauser SL . The charcot lecture | beating MS: a story of B cells, with twists and turns. Multiple Sclerosis J 2015;21:8–21. 10.1177/1352458514561911 PMC4580247 25480864

[2] Hauser SL , Bar‐Or A , Comi G , et al. Ocrelizumab versus Interferon Beta‐1a in relapsing multiple sclerosis. N Engl J Med 2017;376:221–234. 28002679 10.1056/NEJMoa1601277

[3] Montalban X , Hauser SL , Kappos L , et al. Ocrelizumab versus placebo in primary progressive multiple sclerosis. N Engl J Med 2017;376:209–220. 28002688 10.1056/NEJMoa1606468

[4] Salzer J , Svenningsson R , Alping P , et al. Rituximab in multiple sclerosis: A retrospective observational study on safety and efficacy. Neurology 2016;87:2074–2081. 27760868 10.1212/WNL.0000000000003331 PMC5109942

[5] Hauser SL , Waubant E , Arnold DL , et al. B‐Cell depletion with rituximab in relapsing‐remitting multiple sclerosis. N Engl J Med 2008;358(7):676–688. 18272891 10.1056/NEJMoa0706383

[6] Hawker K , O'Connor P , Freedman MS , et al. Rituximab in patients with primary progressive multiple sclerosis: results of a randomized double‐blind placebo‐controlled multicenter trial. Ann Neurol 2009;66:460–471. 19847908 10.1002/ana.21867

[7] Alcalá C , Gascón F , Pérez‐Miralles F , et al. Efficacy and safety of rituximab in relapsing and progressive multiple sclerosis: a hospital‐based study. J Neurol 2018;265:1690–1697. 29785523 10.1007/s00415-018-8899-3

[8] Hu Y , Nie H , Yu H‐H , et al. Efficacy and safety of rituximab for relapsing‐remitting multiple sclerosis: a systematic review and meta‐analysis. Autoimmun Rev 2019;18:542–548. 30844555 10.1016/j.autrev.2019.03.011

[9] Naegelin Y , Naegelin P , von Felten S , et al. Association of rituximab treatment with disability progression among patients with secondary progressive multiple sclerosisrituximab treatment and disability progression in secondary progressive multiple sclerosisrituximab treatment and disability progression in secondary progressive multiple sclerosis. JAMA Neurol 2019;76:274–281. 30615019 10.1001/jamaneurol.2018.4239 PMC6439730

[10] Yamout BI , El‐Ayoubi NK , Nicolas J , et al. Safety and efficacy of rituximab in multiple sclerosis: a retrospective observational study. J Immunol Res 2018;2018:9084759‐. 30539030 10.1155/2018/9084759 PMC6260423

[11] Granqvist M , Boremalm M , Poorghobad A , et al. Comparative effectiveness of rituximab and other initial treatment choices for multiple sclerosis. JAMA Neurol 2018;75:320–327. 29309484 10.1001/jamaneurol.2017.4011 PMC5885857

[12] Boremalm M , Juto A , Axelsson M , et al. Natalizumab, rituximab and fingolimod as escalation therapy in multiple sclerosis. Eur J Neurol 2019;26:1060–1067. 30762259 10.1111/ene.13936

[13] Alping P , Frisell T , Novakova L , et al. Rituximab versus fingolimod after natalizumab in multiple sclerosis patients. Ann Neurol 2016;79(6):950–958. 27038238 10.1002/ana.24651

[14] Scotti B , Disanto G , Sacco R , et al. Effectiveness and safety of Rituximab in multiple sclerosis: an observational study from Southern Switzerland. PLoS One 2018;13:e0197415. 29758075 10.1371/journal.pone.0197415 PMC5951582

[15] Team RDC . R: A language and environment for statistical computing: Vienna, Austria: the R Foundation for Statistical Computing; 2014.

[16] Vollmer B , Nair KV , Sillau SH , et al. Comparison of fingolimod and dimethyl fumarate in the treatment of multiple sclerosis: two‐year experience. Mult Scler J Exp Transl Clin 2017;3:2055217317725102. 28839949 10.1177/2055217317725102 PMC5564884

[17] Vollmer BL , Nair KV , Sillau S , et al. Natalizumab versus fingolimod and dimethyl fumarate in multiple sclerosis treatment. Ann Clin Transl Neurol 2018;6: 252‐262. 30847358 10.1002/acn3.700 PMC6389745

[18] Rubin DB . Using propensity scores to help design observational studies: application to the tobacco litigation. Health Serv Outcomes Res Method 2001;2:169–188.

[19] Giovannoni G , Turner B , Gnanapavan S , et al. Is it time to target no evident disease activity (NEDA) in multiple sclerosis? Mult Scler Relat Disord 2015;4:329–333. 26195051 10.1016/j.msard.2015.04.006

[20] Havrdová E , Arnold DL , Bar‐Or A , et al. No evidence of disease activity (NEDA) analysis by epochs in patients with relapsing multiple sclerosis treated with ocrelizumab vs interferon beta‐1a. Mult Scler J Exper Trans Clin 2018;4:2055217318760642. 10.1177/2055217318760642 PMC5858626 29568544

[21] Nixon R , Bergvall N , Tomic D , et al. No evidence of disease activity: indirect comparisons of oral therapies for the treatment of relapsing‐remitting multiple sclerosis. Adv Ther 2014;31:1134–1154. 25414048 10.1007/s12325-014-0167-z PMC4245493

[22] de Flon P , Laurell K , Söderström L , et al. Improved treatment satisfaction after switching therapy to rituximab in relapsing–remitting MS. Mult Scler J 2017;23:1249–1257. 10.1177/1352458516676643 27780912

[23] Devonshire V , Lapierre Y , Macdonell R , et al. The Global Adherence Project (GAP): a multicenter observational study on adherence to disease‐modifying therapies in patients with relapsing‐remitting multiple sclerosis. Eur J Neurol 2011;18:69–77. 20561039 10.1111/j.1468-1331.2010.03110.x

[24] Tan H , Cai Q , Agarwal S , et al. Impact of adherence to disease‐modifying therapies on clinical and economic outcomes among patients with multiple sclerosis. Adv Ther 2011;28:51–61. 21153000 10.1007/s12325-010-0093-7

[25] Patti F . Optimizing the benefit of multiple sclerosis therapy: the importance of treatment adherence. Patient preference and adherence. 2010;4:1–9. 20165593 10.2147/ppa.s8230 PMC2819898

[26] Richter A , Anton SE , Koch P , Dennett SL . The impact of reducing dose frequency on health outcomes. Clin Ther 2003;25:2307–2335. 14512137 10.1016/s0149-2918(03)80222-9

[27] Christou EAA , Giardino G , Worth A , Ladomenou F . Risk factors predisposing to the development of hypogammaglobulinemia and infections post‐Rituximab. Int Rev Immunol 2017;36:352–359. 28800262 10.1080/08830185.2017.1346092

[28] Barra ME , Soni D , Vo KH , et al. Experience with long‐term rituximab use in a multiple sclerosis clinic. Mult Scler J Exp Transl Clin 2016;2:2055217316672100‐. 28607739 10.1177/2055217316672100 PMC5433395

