# Supplementary material for: Rituximab versus natalizumab, fingolimod, and dimethyl fumarate in multiple sclerosis treatment
Source: Ann Clin Transl Neurol. 2020 Aug 6;7(9):1466–76. doi: 10.1002/acn3.51111 (PMC7480919; doi:10.1002/acn3.51111)
Supplement: Supplementary file 1 — Data S1. SupplementalRTXCOMP.pdf. Supplemental tables and figures examining Cohen’s D values for effect sizes comparing baseline covariates (Figure S1), outcomes for the relapsing‐remitting multiple sclerosis patients only (Table S1‐S3), disease activity during months 6‐24 (Table S4), time to event analyses for effectiveness (Table S5), discontinuations overall excluding insurance issues (Table S6 and Figure S2), discontinuations due to adverse events (Table S7 and Figure S3), and types of adverse events (Table S8). [file ACN3-7-1466-s001.pdf]

Supplemental

Cohen's D Plots

Figure S1. (a) Cohen's D values for effect sizes comparing baseline covariates between NTZ and RTX. (b) Cohen's D values for effect sizes comparing baseline covariates between FTY and RTX. (c) Cohen's D values for effect sizes comparing baseline covariates between DMF and RTX. Logit [PS] : Propensity scores generated through logistic regression modeled the probability of receiving RTX treatment using the pre-selected covariates of age, sex, disease duration, diagnosis and CEL on baseline MRI

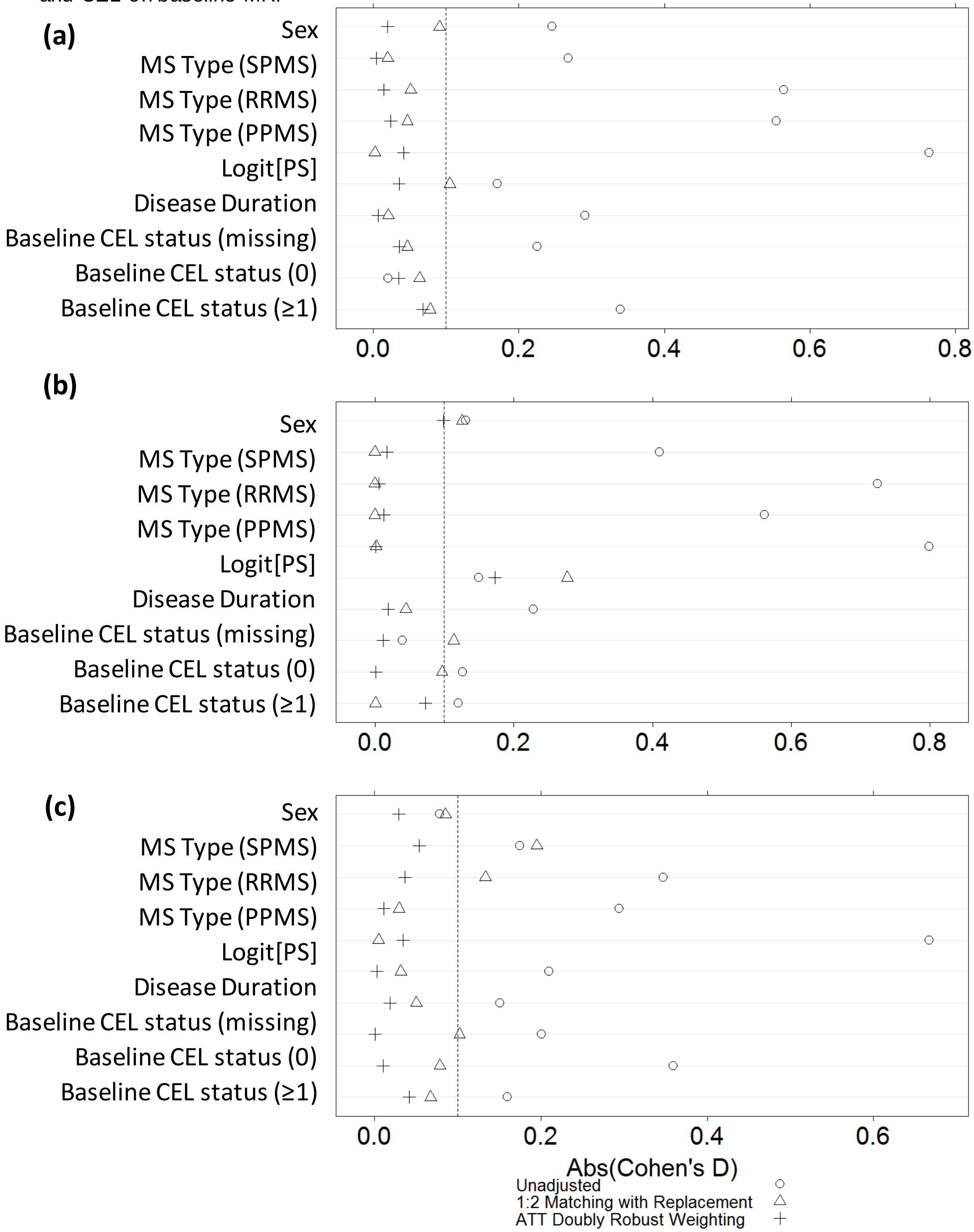

Relapsing-Remitting Multiple Sclerosis Cohort

Table S1. Baseline characteristics for rituximab (RTX), natalizumab (NTZ), fingolimod (FTY) and dimethyl fumarate (DMF) study cohorts for RRMS patients.

	Rituximab (N=113)	Natalizumab (N=382)		Fingolimod (N=244)		Dimethyl Fumarate (N=265)	
	**N or Mean (SD or %)**	**N or Mean (SD or %)**	**p-value^**	**N or Mean (SD or %)**	**p-value^**	**N or Mean (SD or %)**	**p-value^**
**Disease Duration (Years, SD)**	11.2 (7.3)	10.5 (6.7)	0.353	10.4 (6.3)	0.291	9.9 (6.6)	0.110
**Age (Years, SD)**	41.3 (11.8)	37.9 (11.3)	**0.007**	41.5 (11.2)	0.896	43.5 (12.0)	0.096
**Gender - Female**	80 (70.8%)	298 (78.0%)	0.113	178 (73.0%)	0.672	192 (72.5%)	0.743
**Previous DMT***			**<0.001**		**<0.001**		**<0.001**
Interferons	2 (1.8%)	82 (21.5%)		35 (14.3%)		39 (14.7%)	
Glatiramer acetate	10 (8.9%)	140 (36.7%)		46 (18.9%)		91 (34.3%)	
Natalizumab	60 (53.1)	0 (0.0%)		97 (39.8%)		48 (18.1%)	
Rituximab	0 (0.0%)	0 (0.0%)		1 (0.4%)		5 (1.9%)	
Fingolimod	16 (14.2%)	7 (1.8%)		0 (0.0%)		17 (6.4%)	
Dimethyl fumarate	0 (0.0%)	2 (0.5%)		1 (0.4%)		0 (0.0%)	
None	24 (21.2%)	141 (36.9%)		61 (25.0%)		60 (22.6%)	
Other	1 (0.9%)	10 (2.6%)		3 (1.2%)		4 (1.5%)	
**Contrast Enhancement on Baseline MRI**	31 (30.1%)	112 (35.3%)	0.330	53 (25.5%)	0.388	37 (15.9%)	**0.003**
**Disease Burden on Baseline MRI$^{\beta}$**			**<0.001**		**0.009**		**<0.001**
Mild	31 (27.1%)	177 (46.3%)		92 (37.7%)		140 (52.8%)	
Moderate	47 (41.6%)	105 (27.5%)		70 (27.7%)		71 (26.8%)	
Severe	24 (21.2%)	25 (6.5%)		37 (15.2%)		16 (6.0%)	
Missing	11 (9.7%)	75 (19.6%)		45 (18.4%)		38 (14.3%)	

RRMS: relapsing-remitting multiple sclerosis
^ In comparison to RTX
* Within 6 months prior to starting study drug
$^{\beta}$Disease burden at baseline is defined as mild < 10 T2/Flair Lesions, moderate 10-20 T2/FLAIR lesions, severe > 20 T2/FLAIR lesions"
Bold p-values indicate p>0.05 and are considered statistically significant.

Table S2. Unadjusted & adjusted odds ratios for disease activity regardless of discontinuation in a composite effectiveness measure (new T2 lesion, CEL, and/or new clinical relapse) at ≤24 months in RRMS patients.

	NTZ vs RTX			FTY vs RTX			DMF vs RTX		
	**N**	**Odds Ratio** (95 % CI)	**p-value**	**N**	**Odds Ratio** (95 % CI)	**p-value**	**N**	**Odds Ratio** (95 % CI)	**p-value**
**Simple Logistic Regression**	495	**1.65** (0.94, 2.88)	0.078	357	**2.98** (1.69, 5.26)	**<0.001**	378	**3.15** (1.79, 5.53)	**<0.001**
**Adjusted Logistic Regression***	495	**1.54** (0.87, 2.73)	0.135	357	**3.36** (1.87, 6.05)	**<0.001**	378	**3.97** (2.15, 7.31)	**<0.001**
**ATT Doubly Robust Weighting Estimator***	495	**1.58** (0.90, 2.76)	0.085	357	**3.19** (1.82, 5.57)	**<0.001**	378	**3.56** (2.03, 6.23)	**<0.001**

NTZ: natalizumab; RTX: rituximab; FTY: fingolimod; DMF: dimethyl fumarate; CEL: contrast enhancing lesion; CI: confidence interval; PM: propensity matching; NN: nearest neighbor; RRMS: relapsing-remitting multiple sclerosis
* controlling for age, disease duration, type of MS, gender, and contrast enhancement on baseline MRI
Bold p-values indicate p>0.05 and are considered statistically significant.

Table S3. Unadjusted & adjusted odds ratios for discontinuation for any reason at ≤24 months in RRMS

	NTZ vs RTX			FTY vs RTX			DMF vs RTX		
	**N**	**Odds Ratio** (95 % CI)	**p-value**	**N**	**Odds Ratio** (95 % CI)	**p-value**	**N**	**Odds Ratio** (95 % CI)	**p-value**
**Simple Logistic Regression**	495	**1.57** (0.96, 2.58)	0.073	357	**1.69** (1.00, 2.83)	**0.049**	378	**2.96** (1.78, 4.91)	**<0.001**
**Adjusted Logistic Regression***	495	**1.45** (0.87, 2.41)	0.155	357	**1.77** (1.05, 2.99)	**0.033**	378	**3.45** (2.02, 5.89)	**<0.001**
**ATT Doubly Robust Weighting Estimator***	495	**1.47** (0.89, 2.44)	0.118	357	**1.73** (1.04, 2.90)	**0.026**	378	**3.10** (1.68, 5.69)	**<0.001**

NTZ: natalizumab; RTX: rituximab; FTY: fingolimod; DMF: dimethyl fumarate; CEL: contrast enhancing lesion; CI: confidence interval; PM: propensity matching; NN: nearest neighbor; RRMS: relapsing-remitting multiple sclerosis
* controlling for age, disease duration, type of MS, gender, and contrast enhancement on baseline MRI
Bold p-values indicate p>0.05 and are considered statistically significant.

Odds of Experiencing Disease Activity between 6-24 Months of Treatment

All Subjects on Therapy >6 Months Included in Analysis

Table S4. Unadjusted & adjusted odds ratios for disease activity regardless of discontinuation in a composite effectiveness measure (new T2 lesion, CEL, and/or new clinical relapse) between 6-24 Months of treatment.

	NTZ vs RTX			FTY vs RTX			DMF vs RTX		
	**N**	**Odds Ratio** (95 % CI)	**p-value**	**N**	**Odds Ratio** (95 % CI)	**p-value**	**N**	**Odds Ratio** (95 % CI)	**p-value**
**Simple Logistic Regression**	599	**2.28** (1.31, 3.98)	**0.004**	416	**3.61** (2.03, 6.43)	**<0.001**	462	**2.79** (1.57, 4.95)	**<0.001**
**Adjusted Logistic Regression***	599	**2.27** (1.27, 4.07)	**0.006**	416	**4.07** (2.05, 8.06)	**<0.001**	462	**3.13** (1.63, 5.99)	**0.001**
**PM 1:2 NN with replacement***	543 374 unique	**2.31** (1.22, 4.39)	**0.010**	543 338 unique	**3.48** (1.65, 7.36)	**0.001**	543 345 unique	**2.56** (1.32, 4.99)	**0.006**
**ATT Doubly Robust Weighting Estimator***	599	**2.21** (1.20, 4.06)	**0.007**	416	**4.25** (2.16, 8.36)	**<0.001**	462	**3.10** (1.68, 5.69)	**<0.001**

NTZ: natalizumab; RTX: rituximab; FTY: fingolimod; DMF: dimethyl fumarate; CEL: contrast enhancing lesion; CI: confidence interval; PM: propensity matching; NN: nearest neighbor

* controlling for age, disease duration, type of MS, gender, and contrast enhancement on baseline MRI

Bold p-values indicate p>0.05 and are considered statistically significant.

Time to Event Analysis of Composite Effectiveness Measure for Entire Cohort

Table S5. Time to event analyses for composite disease activity measure.

**Model Type (NTZ vs RTX)**	**Adjustment**	**Hazard Ratio (95% CI)**	**p-value**
State Transition (Population)	None	1.58 (1.05, 2.40)	**0.030**
State Transition (Population)	Full*	1.49 (0.96, 2.30)	0.077
**Model Type (FTY vs RTX)**	**Adjustment**	**Hazard Ratio (95% CI)**	**p-value**
State Transition (Population)	None	2.81 (1.86, 4.27)	**<0.001**
State Transition (Population)	Full*	3.18 (1.99, 5.07)	**<0.001**
**Model Type (DMF vs RTX)**	**Adjustment**	**Hazard Ratio (95% CI)**	**p value**
State Transition (Population)	None	2.97 (1.98, 4.45)	**<0.001**
State Transition (Population)	Full*	3.38 (2.20, 5.20)	**<0.001**

*controlling for age, disease duration, type of MS, gender, and contrast enhancement on baseline MRI

Bold p-values indicate p>0.05 and are considered statistically significant.

Discontinuation for Any Reason Other Than Insurance

Table S6. Unadjusted & adjusted odds ratios for discontinuation for any reason other than insurance at ≤24 months.

	NTZ vs RTX			FTY vs RTX			DMF vs RTX		
	**N**	**Odds Ratio** (95 % CI)	**p-value**	**N**	**Odds Ratio** (95 % CI)	**p-value**	**N**	**Odds Ratio** (95 % CI)	**p-value**
**Simple Logistic Regression**	633	**2.25** (1.43, 3.55)	**<0.001**	453	**2.44** (1.51, 3.94)	**<0.001**	524	**4.09** (2.58, 6.48)	**<0.001**
**Adjusted Logistic Regression***	633	**2.33** (1.43, 3.79)	**0.001**	453	**3.12** (1.84, 5.31)	**<0.001**	524	**5.65** (3.40, 9.38)	**<0.001**
**PM 1:2 NN with replacement***	546 372 Unique	**2.34** (1.33, 4.11)	**0.003**	546 347 unique	**3.48** (1.80, 6.76)	**<0.001**	546 371 unique	**5.15** (2.96, 8.95)	**<0.001**
**ATT Doubly Robust Weighting Estimator***	633	**2.36** (1.44, 3.88)	**<0.001**	453	**2.97** (1.71, 5.17)	**<0.001**	524	**5.22** (3.27, 8.34)	**<0.001**

NTZ: natalizumab; RTX: rituximab; FTY: fingolimod; DMF: dimethyl fumarate; CI: confidence interval; PM: propensity matching; NN: nearest neighbor.
* controlling for age, disease duration, type of MS, gender, and contrast enhancement on baseline MRI
Bold p-values indicate p>0.05 and are considered statistically significant.

Figure S2. Kaplan-Meier failure curves demonstrating cumulative probability of discontinuation for any reason other than insurance over time.

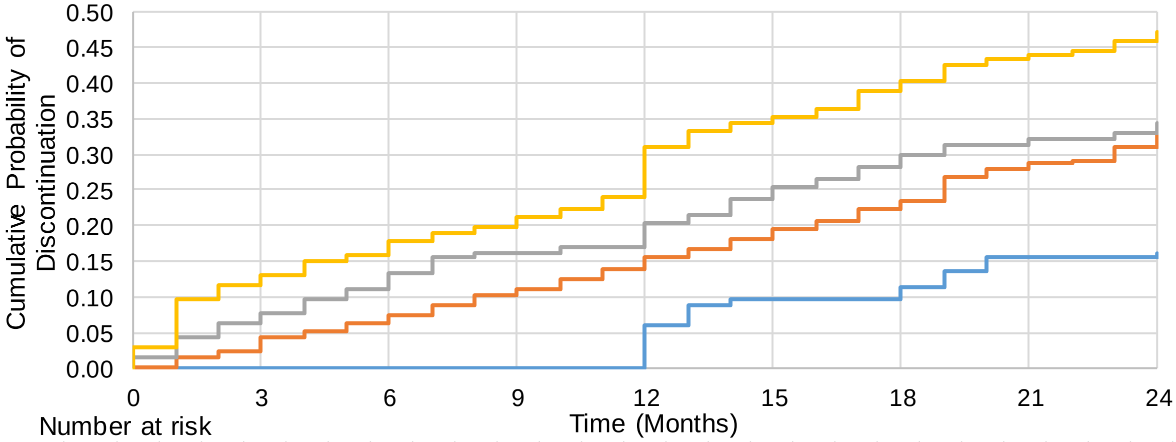

Number at risk

RTX	182	182	182	182	182	182	182	181	180	180	180	179	179	167	160	159	157	154	154	149	142	139	139	137	137
NTZ	451	450	444	440	431	428	422	418	411	405	401	395	388	381	376	370	363	358	351	345	331	325	322	320	312
FTY	271	267	259	254	250	245	241	235	229	227	227	225	225	216	213	207	202	199	195	190	186	186	184	184	182
DMF	342	332	309	302	297	291	288	281	277	274	270	266	260	236	228	224	222	218	209	204	197	194	192	190	185

Rituximab — Natalizumab — Fingolimod — Dimethyl Fumarate

Table S7. Unadjusted & adjusted odds ratios for discontinuation due to adverse events at ≤24 months.

	NTZ vs RTX			FTY vs RTX			DMF vs RTX		
	**N**	**Odds Ratio** (95 % CI)	**p-value**	**N**	**Odds Ratio** (95 % CI)	**p-value**	**N**	**Odds Ratio** (95 % CI)	**p-value**
**Simple Logistic Regression**	633	**1.01** (0.51, 2.02)	0.979	453	**2.90** (1.49, 5.64)	**0.002**	524	**4.47** (2.36, 8.44)	**<0.001**
**Adjusted Logistic Regression***	633	**0.92** (0.44, 1.90)	0.819	453	**3.21** (1.63, 6.33)	**0.001**	524	**6.09** (3.09, 11.99)	**<0.001**
**PM 1:2 NN with replacement***	546 372 Unique	**0.91** (0.40, 2.10)	0.827	546 347 unique	**3.55** (1.62, 7.78)	**0.002**	546 371 unique	**3.31** (1.63, 6.74)	**0.001**
**ATT Doubly Robust Weighting Estimator***	633	**0.793** (0.39, 1.63)	0.545	453	**2.65** (1.30, 5.37)	**0.004**	524	**4.98** (2.57, 9.65)	**<0.001**

NTZ: natalizumab; RTX: rituximab; FTY: fingolimod; DMF: dimethyl fumarate; CI: confidence interval; PM: propensity matching; NN: nearest neighbor.
* controlling for age, disease duration, type of MS, gender, and contrast enhancement on baseline MRI
Bold p-values indicate p>0.05 and are considered statistically significant.

Figure S3. Kaplan-Meier failure curves demonstrating cumulative probability of discontinuation due to adverse events

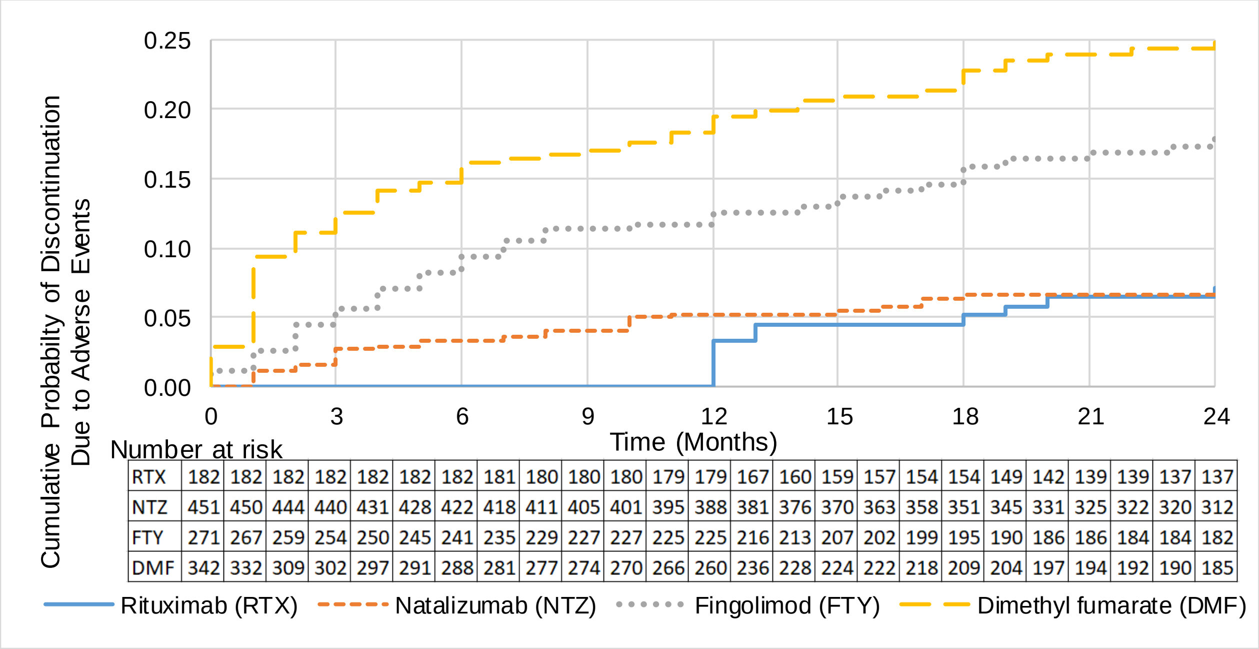

RTX	182	182	182	182	182	182	182	181	180	180	180	179	179	167	160	159	157	154	154	149	142	139	139	137	137
NTZ	451	450	444	440	431	428	422	418	411	405	401	395	388	381	376	370	363	358	351	345	331	325	322	320	312
FTY	271	267	259	254	250	245	241	235	229	227	227	225	225	216	213	207	202	199	195	190	186	186	184	184	182
DMF	342	332	309	302	297	291	288	281	277	274	270	266	260	236	228	224	222	218	209	204	197	194	192	190	185

Table S8. Percentage of all patients on indicated therapy who experienced adverse events leading to discontinuation.

Adverse Event	Rituximab N=182		Natalizumab N=451		Fingolimod N=271		Dimethyl fumarate N=342	
	N	%	N	%	N	%	N	%
Infections	5	2.7%	3	0.7%	7	2.6%	4	1.2%
Neutropenia	3	1.6%	-	-	-	-	-	-
Shortness of Breath (NAb+)	2	1.1%	2 (2)	0.4% (0.4%)	3	1.1%	-	-
Worsening Sarcoidosis	1	0.5%	-	-	-	-	-	-
Low Platelet Count	1	0.5%	-	-	-	-	-	-
GI Issues	--	-	4	0.9%	11	4.1%	66	19.3%
Flushing/Rash/Hot flashes (NAb+)	-	-	14 12	3.1% (2.7%)	-	-	25	7.3%
Headaches	-	-	5	1.1%	8	3.0%	1	0.3%
Fatigue	-	-	6	1.3%	-	-	-	-
Lymphopenia	-	-	-	-	7	2.6%	6	1.8%
Elevated LFTs	-	-	3	0.7%	6	2.2%	1	0.3%
Psychiatric disorders	-	-	4	0.9%	2	4.3%	1	0.3%
Weight Gain	-	-	1	0.2%	1	0.4%	1	0.3%
Hypotension	-	-	1	0.2%	-	-	-	-
Ovarian Cyst	-	-	1	0.2%	-	-	-	-
Arrhythmia	-	-	-	-	4	1.5%	-	-
Hair Loss	-	-	-	-	2	0.7%	2	0.6%
Bradycardia	-	-	-	-	3	1.1%	-	-
Hypertension	-	-	-	-	3	1.1%	-	-
Tachycardia	-	-	-	-	3	1.1%	-	-
Muscle Spasms/weakness	-	-	1	0.2%	1	0.4%	3	0.9%
Taste & vision changes	-	-	-	-	2	0.7%	1	0.3%
Reported pain (Other than Abdominal)	-	-	-	-	1	0.4%	2	0.6%
Alveolar Hemorrhage	-	-	-	-	1	0.4%	-	-
Palpitations	-	-	-	-	1	0.4%	-	-
Pancytopenia	-	-	-	-	1	0.4%	-	-
Seizures	-	-	-	-	1	0.4%	-	-

NAb+: neutralizing antibody positive; GI: gastrointestinal; LFTs: liver function test.
Note: Discontinuation occurred in 46 RTX, 147 NTZ, 93 FTY, and 161 DMF patients.
Discontinuations due to AEs occurred in 12 RTX, 30 NTZ, 46 FTY, and 82 DMF patients.
